# TECHNOLOGY TRANSLATING THE RELATIONSHIP BETWEEN QUALITY OF LIFE AND MEMORY USING A NOVEL EEG TECHNOLOGY

**DOI:** 10.1093/geroni/igz038.1207

**Published:** 2019-11-08

**Authors:** Mitchell Roberts, Orli Kehat, Michaela Gross, Nethanel Lasri, Gil Issachar, Erica Sappington VandeWeerd, Ziv Peremen, Amir Geva

**Affiliations:** 1 University of South Florida, Tampa, Florida, United States; 2 elminda, Hertsliya, Israel

## Abstract

Existing research has postulated a relationship between cognition and quality of life (QoL). Components of QoL such as satisfaction with social support may be particularly influential in memory for those with comorbidities. Additional research is needed to characterize the relationship between memory and QoL domains. Findings are presented from a clinical trial using BNA memory scores to assess brain health. BNA uses EEG technology and machine learning to map networks of brain functioning including working memory. Participants were older adults living in The Villages, an active lifestyle community in Florida, between the ages of 55-85, from 8/30/2017-3/11/2019. Participants were stratified into 2 groups: healthy (no CNS/psychiatric conditions; n=158) and multi-morbid (>1 CNS and/or psychiatric conditions; n=106) and compared across memory and QoL indicators. Subjective QoL was measured by the WHOQOL-BREF across 4 domains (physical, psychological, social, environmental). Scores on QoL domains were divided into 3 levels (high-medium-low) and tested for their relationship to BNA memory scores using ANOVA. Results indicate a relationship between health status, subjective QoL and BNA memory scores. Healthy subjects who scored high in the psychological QoL domain had significantly higher memory scores [F(2,152)=4.30,p=.02)]. In healthy subjects, satisfaction with social support (p=.001) had the strongest impact on memory for social QoL, while body image (p=.06) and concentration (p=.06) were the most salient predictors of psychological QoL and approached significance. Multi-morbid subjects who indicated high social ratings had higher memory scores (F(2,100)=3.75,p=.03) which relied heavily on satisfaction with social support (p=.003). Implications for policy and practice are discussed.

